# Fate and Biofilm Formation of Wild-Type and Pressure-Stressed Pathogens of Public Health Concern in Surface Water and on Abiotic Surfaces

**DOI:** 10.3390/microorganisms8030408

**Published:** 2020-03-13

**Authors:** Md Niamul Kabir, Sadiye Aras, Sabrina Wadood, Shahid Chowdhury, Aliyar Cyrus Fouladkhah

**Affiliations:** 1 Public Health Microbiology Laboratory, Tennessee State University, Nashville, TN 37209, USA; mkabir@my.tnstate.edu (M.N.K.); saras@my.tnstate.edu (S.A.); swadood@tnstate.edu (S.W.); schowdh1@tnstate.edu (S.C.); 2 Cooperative Extension Program, Tennessee State University, Nashville, TN 37209, USA

**Keywords:** biofilm formation, pressure-stressed bacteria, *Listeria monocytogenes*, non-typhoidal *Salmonella enterica* serovars, Shiga toxin-producing *Escherichia coli* O157:H7, surface water

## Abstract

Since the historic outbreak near Broad Street in London, which serves as cornerstone of modern epidemiology, infectious diseases spread in surface and sub-surface water has been a persisting public health challenge. The current study investigated persistence of wild-type and pressure-stressed *Listeria monocytogenes*, *Escherichia coli* O157:H7, and non-typhoidal *Salmonella enterica* serovars in surface water stored aerobically for up to 28 days at 5, 25, and 37 °C. Additionally, biofilm formation of wild-type and pressure-stressed non-typhoidal *Salmonella* serovars were monitored on surface of stainless steel and rubber coupons for 28 days at 25 and 37 °C. While *L. monocytogenes* exhibited a lower (*p* < 0.05) survival rate at 5 °C, relative to the two Gram-negative pathogens, at higher temperatures of 25 and 37 °C, all three pathogens exhibited similar (*p* ≥ 0.05) trends for survival in surface water. Both wild-type and pressure-stressed *Salmonella* serovars in the vast majority of tested times, temperatures, and surfaces exhibited comparable (*p* ≥ 0.05) persistence and biofilm formation capability. Our study thus indicates the occurrence of contamination could lead to prolonged survival of these microorganisms in low-nutrient environments and highlights the need for preventive measures such as those articulated under Produce Safety Rule of the U.S. Food Safety Modernization Act.

## 1. Introduction

Since the historic 1854 water outbreak near Broad Street in Soho district of Westminster in London, that serves as the cornerstone for modern epidemiology [1], safety of water supplies has been an integral part of public health practices around the globe. The changing climate additionally serves as a “threat multiplier,” and is expected to accelerate the spread of waterborne infectious diseases and subsequently food safety issues due to increases in extreme weather events and acceleration in multiplication of bacterial pathogens [2,3]. The presence of pathogenic bacteria of public health concern in private and public water systems has arisen in the last few years, serving as etiological agents for an array of infectious diseases and outbreaks nationally and globally associated with raw agricultural commodities. Agricultural water is considered as one of the most important risk factors for contamination of fresh produce and raw agricultural commodities [4,5] and has recently been regulated in the United States under the Produce Safety Rule of Food Safety Modernization Act. The legislation is imposing water safety/testing regulatory requirements for growers of raw agricultural commodities for the first time in the history of the country [2].

The epidemiological data derived from the U.S. Centers for Disease Control and Prevention’s (CDC) National Outbreak Reporting System (NORS) active surveillance data indicates that from 2009 to 2017, at least 743 waterborne outbreaks occurred in the United States and among them 15 occurred by *Escherichia coli* O157:H7 and/or non-typhoidal *Salmonella* serovars [6]. Due to their presence in surface water reservoirs and involvement in recent outbreaks, *Listeria monocytogenes* have also been gaining increasing attention as waterborne pathogens [7]. Surveys of different water bodies in different regions of the United States and abroad had reported the presence of different human pathogens [8]. Based on the survey study on rural surface water and watershed in south central Georgia in the United States, it has been reported that, in surface water, the prevalence of *Salmonella* serovars could be as high as 62% [9,10]. Another study conducted in 2012 from 27 sites in North America (total of 902 samples) exhibited that 80% of collected water samples could carry human pathogens, such as pathogenic serogroups of *Escherichia coli* [11].

In addition to wild-type phenotype of a microorganism, microbial pathogens exposed to a stressor might have accelerated or diminished sensitivity to subsequent stressor(s). A stressor that could be physical, chemical, or biological in nature (such as cold and heat shock, oxidative or acid responses) could lead to cross-protection of the pathogen to a subsequent hurdle or could enhance the sensitivity of the pathogen to subsequent stressors [12]. While the effects of an array of stressors are investigated and reviewed in the literature [12], there is currently a knowledge gap for the assimilation of the effects of sub-lethal exposure to elevated hydrostatic pressure on the fate and biofilm formation of an array of microbial pathogens. This is of particular importance since the utilization of elevated hydrostatic pressure is gaining increasing importance and momentum in food manufacturing [13,14,15,16]. Information about the survival and biofilm formation of the pressure-stressed phenotype of the main microbial pathogens is critical to assure existing validation studies conducted using wild-type phenotypes are capable of controlling the pressure-stressed phenotypes of the pathogens as well. 

As the leading cause of foodborne hospitalization and death in the United States, in addition to proliferation capability, non-typhoidal *Salmonella* serovars are capable of forming sessile biofilm communities that could, to a great extent, enhance their persistence and response to environmental stressors [17]. As such, the first objective of the current study was to determine the persistence and proliferation of wild-type and pressure-stressed phenotypes of *E. coli* O157:H7, *L. monocytogenes,* and non-typhoidal *Salmonella* serovars in surface water at 5, 25, and 37 °C for 28 days. Additionally, biofilm formation of *Salmonella enterica* serovars on two abiotic surfaces were investigated during 28-day period at 25 and 37 °C.

## 2. Materials and Methods

### 2.1. Planktonic Cells Preparations and Enumeration

A four-strain mixture of *L. monocytogenes* (ATCC® numbers 51772, 51779, BAA-2657, and 13932), six-strain mixture of *E. coli* O157:H7 (ATCC® numbers BAA-460, 43888, 43894, 35150, 43889, 43890), and five-strain mixture of non-typhoidal *Salmonella enterica* serovars (ATCC® numbers 13076, 8387, 6962, 9270, 14028) were used in this study. The strains are selected based on preliminary data and their epidemiological and public health significance that are elaborated in detail in our recent open access publications [13,14,15,16]. These strain mixtures were used for inoculation of Cumberland river (Nashville, TN) water samples that were autoclaved for 15 min at 121 °C (MLS3781L-PA model, PHC Corporation of North America, Wood Dale, IL, USA). All bacterial strains were originally obtained from American Type Culture Collection and were available in Public Health Microbiology laboratory of Tennessee State University (Nashville, TN). 

For each strain separately, the cultures of *L. monocytogenes, E. coli* O157:H7, and *Salmonella* serovars were prepared on yeast extract (0.6%) supplemented Tryptic Soy Agar (TSA + YE) (Difco, Becton Dickinson, Franklin Lakes, NJ, USA) and plates incubated for 24 h at 37 °C. Two days before the experiment trials, each strain of *L. monocytogenes, E. coli* O157:H7, and *Salmonella* serovars were individually activated into 10 mL of Tryptic Soy Broth (Difco, Becton Dickinson, Franklin Lakes, NJ, USA) supplemented with 0.6% yeast extract (TSB + YE) by aseptically transferring a loopful of single colonies incubated at 37 °C for 20–24 h [14]. After incubation for 24 h, 100 µL aliquots of the activated culture suspension were individually and aseptically sub-cultured into another 10 mL of TSB + YE for 22–24 h at 37 °C. Each overnight sub-cultured strain (2 mL per strain) was then harvested by centrifugation (Model 5424, Eppendorf North America, Hauppauge, NY, USA; Rotor FA-45-24-11) at 6000 revolutions per minute (RPM) for 15 min (3548 g, 88 mm rotor). This step was repeated twice by re-suspending the bacterial pellets, for each strain individually, in Phosphate Buffered Saline (PBS, VWR International, Radnor, PA, USA) to remove excreted secondary metabolites, growth medium, and sloughed cell components. Three separate mixture of *L. monocytogenes, E. coli* O157:H7 and *Salmonella* serovars were then obtained by combining re-suspended bacterial cultures into sterilized water samples.

### 2.2. Biofilm Fate and Formation 

New and previously used stainless steel (type 304, #2b) coupons (total surface area 15.51 cm^2^) and rubber (Ethylene Propylene Diene Monomer) coupons (total surface area of 26.82 cm^2^) were used in this study. Coupons were cleaned with soap, water, 99% acetone (Fischer Scientific, Fair Lawn, N.J., USA) for 5 min, and 70% ethyl alcohol (Fischer Scientific) for 5 min, to remove any residues, and autoclaved using the above-mentioned time, intensity, and instrument [17,18]. Sterile and dry coupons were spot-inoculated (0.1 mL per coupon) using the above-referenced (Section 2.1.) *Salmonella* serovars bacterial suspension. Additionally, a separate set of coupons were inoculated with 0.1 mL of pressure-stressed *Salmonella* serovars suspension. The pressure-stressed phenotype was prepared by exposing the bacterial suspension to 15,000 PSI (103 MPa) of elevated hydrostatic pressure at 4 °C for 15 min (Barocycler Hub880, Pressure BioScience Inc., South Easton, MA, USA). Temperature values were monitored during the pressure treatment by a T-type thermocouple and controlled by a circulating water bath connected to a stainless steel jacket surrounding the chamber, as further delineated in our open access article [13]. The attachment time of the bacterial cells (wild-type and pressure-stressed) onto the surface of coupons was 1 h under a biosafety cabinet, based on preliminary trials conducted in our previous study [17]. The inoculated coupons were aseptically transferred into 50 mL sterile Eppendorf conical tubes containing 15 mL of autoclaved water samples. Coupons were half-submerged in water and incubated under static and aerobic conditions at 25 and 37 °C for 0 to 28 days. According to a past study, the upper part of the half-submerged coupons is exposed to an air-liquid interface that enhances microbial biofilm formation [17,18]. Tubes were incubated at 25 and 37 °C for both wild-type and pressure-stressed phenotypes, for 0, 1, 4, 7, 8, 11, 14, 21, and 28 days. For investigating the formation of biofilms at 25 and 37 °C, 2 h after inoculation (day 0) and on days 1, 4, 7, 8, 11, 14, 21, and 28, inoculated coupons were analyzed for microbial populations. Recovery of the biofilm mass from the coupons was achieved by first rinsing each side of the submerged coupons with 10 mL of sterile deionized water to remove loosely attached cells. The coupons were then submerged in 30 mL of D/E neutralizing broth solution. Biofilm cells from stainless steel coupons were separated using 20 sterilized glass beads (4 mm diameter) and by vortexing for 2 min (3200 RPM) prior to enumeration [17,19,20]. Biofilm mass of rubber coupons were separated using sonication (40 kHz, Fisherbrand™ CPXH Series Heated Ultrasonic Bath) for 5 min. Previous trials conducted by our research group had indicated the beads method and sonication for 5 min have similar efficacy for removal of bacterial biofilms from surface of coupons [21].

### 2.3. Neutralization, and Microbiological and pH Analyses 

Samples were 10-fold serially diluted in Maximum Recovery Diluent (Difco, Becton Dickinson, Franklin Lakes, NJ, USA) to enhance the recovery of the injured but viable cells and neutralized using D/E neutralizing broth (Difco, Becton Dickinson, Franklin Lakes, NJ, USA). The neutralized diluents were then plated on TSA + YE and additionally for biofilm formation on XLD Agar (Criterion Dehydrated Culture Media CA, USA) for selective *Salmonella* serovars enumeration. All media were then incubated at 37 °C for 24–48 h. Microbial colonies were then manually counted and converted to log values for further descriptive and inferential statistical analyses. The pH values of all enumerated samples were measured, using a digital pH meter calibrated at pH of 4.00, 7.01, and 10.01 before analyses (Mettler Toledo, AG, Switzerland). Relative humidity and temperature of samples during storage and incubation were monitored using a RHTemp101A device (Madge Tech, Wamer, NH, USA).

### 2.4. Experimental Design and Statistical Analyses

As discussed in the introduction section, there were two separate trials in this study. The first set of trails (results illustrated in Figure of Section 3.1) investigated the persistence of *L. monocytogenes*, *E. coli* O157:H7, and *Salmonella* serovars for up to 28 days at 5, 25, and 37 °C. The second trial was conducted to investigate the persistence and biofilm formation of *Salmonella* serovars for up to 28 days at 25 and 37 °C on the surface of stainless steel and rubber coupons (results illustrated in Figures of Section 3.3). All trials were complete randomized block designs consisting of three biologically independent repetitions as blocking factors (i.e., three blocks). Each block additionally consisted of two replications, with each replication consisting of two microbiological repetitions. Therefore, each presented value is the mean of 12 independent observations (i.e., 3 blocks, 2 replications, and 2 microbiological repetitions). Initial data management, log conversions, and a further descriptive analysis of the data was conducted by using Microsoft Excel. Analyses of variance of data were performed using the general linear model procedure of SAS_9.4_ (SAS Institute, Cary, NC, USA), followed by Tukey- and Dunnett’s-adjusted means separation at type I error level of 5%. 

## 3. Results and Discussion

**Figure 1:**
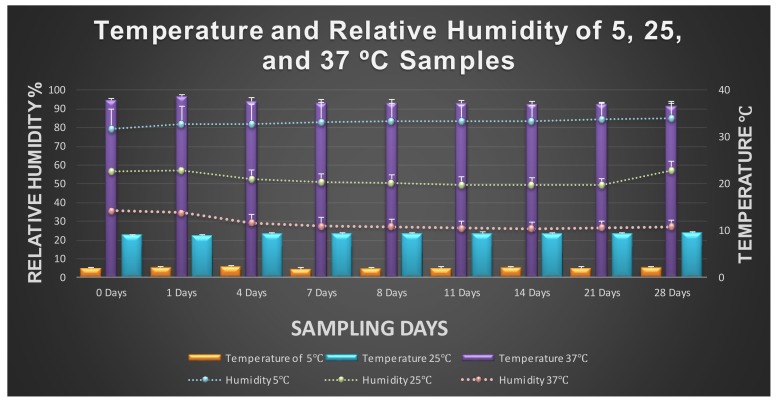
Average hourly temperature and relative humidity of 5, 25, and 37 °C samples during the sampling period from 0 to 28 days. Bar graphs represent temperature and line graphs represent relative humidity.

Since temperature and relative humidity play crucial roles in the persistence and multiplication of bacterial pathogens [13], this study was conducted under close monitoring conditions of these extrinsic factors. For the trials conducted under the target temperature of 5 °C, the temperature values were ranging from 4.46 to 5.59 °C (standard deviations of samples ranging from 0.39 to 0.92). The relative humidity (RH) of the samples at this temperature were 79.16% to 84.86% (standard deviations ranging from 8.10 to 10.62). Trials at a target temperature of 25 °C had temperature values ranging from 22.54 to 23.66 (standard deviations ranging from 0.09 to 0.75) and relative humidity of 49.22 to 57.27 (standard deviations ranging from 0.68 to 5.14). The experiments at a target temperature of 37 °C were conducted under temperature values ranging from 36.51 to 38.56 (standard deviations range 0.44 to 1.13) and under relative humidity ranging from 26.04 to 35.54 (standard deviations ranging from 0.19 to 4.66). These recorded extrinsic values are illustrated in a combination graph (Figure 1). Relative humidity is an important extrinsic factor for multiplication of bacterial pathogens. Typically, an RH of > 60% is an optimum breeding ground for multiplication of bacterial pathogens, while an RH < 20% could inhibit the growth of an array of bacteria [22].

### 3.1. Fate of Planktonic Pathogens at 5 °C

**Figure 2:**
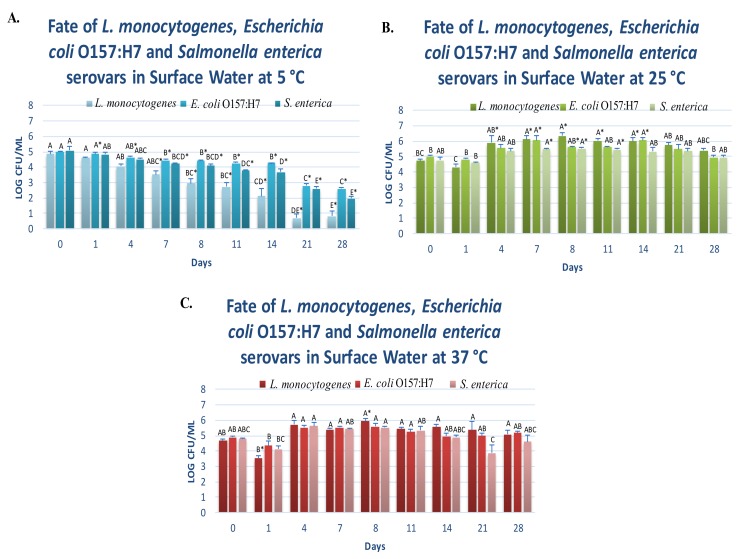
Fate and survival of four-strain cocktail of *Listeria monocytogenes* (ATCC® numbers 51772, 51779, BAA-2657, and 13932), six-strain cocktail of *E. coli* O157:H7 (ATCC® numbers BAA-460, 43888, 43894, 35150, 43889, 43890), and five-strain cocktail of non-typhoidal *Salmonella enterica* serovars (ATCC® numbers 13076, 8387, 6962, 9270, 14028) in autoclaved Cumberland river water for 0, 1, 4, 7, 8, 11, 14, 21, and 28 days at 5, 25, and 37°C. In each graph, and for each pathogen separately, columns of each time interval followed by different uppercase letters are representing log CFU/mL values that are statistically (*p* < 0.05) different (Tukey-adjusted ANOVA). Uppercase letters followed by * sign are statistically (*p* < 0.05) different than the untreated control (Dunnett’s-adjusted ANOVA). (**A**) Survival of three pathogens at 5 °C; (**B**) Survival of three pathogens at 25 °C; (**C**) Survival of three pathogens at 37 °C.

Persistence and proliferation of *L. monocytogenes* were monitored at this temperature for 28 days. As articulated in Section 2.4, this study was conducted in three biologically independent repetitions, each constructed in two replications and an additional two microbiological repetitions. Counts of the pathogen on day zero were 4.86 ± 0.1 log CFU/mL (mean ± SE is reported for this and remaining microbiological observations). After eight days, the *L. monocytogenes* counts were reduced (*p* < 0.05) by 1.92 log CFU/mL (i.e., > 90% reduction). The counts after two weeks of static aerobic storage were similar (*p* ≥ 0.05) to day eight and were 2.13 ± 0.5 (Figure 2A). After the 28-day trial, the counts of *L. monocytogenes* were still above the detection limit and were 0.83 ± 0.3. The reduction (*p* < 0.05) of this Gram-positive pathogen at this temperature was overall 4.03 log CFU/mL (i.e., >99.99% reduction) during the 28-day trial, yet the pathogen remained viable and detectable at this low temperature. It is important to note that *L. monocytogenes* are among very few foodborne pathogens that could multiply under refrigeration temperatures [22]. Our study indicates this pathogen could remain detectable in surface water samples at very low temperatures for several weeks. This is of particular importance for the safety of raw agricultural commodities and those products that could be considered as ready-to-eat. As further delineated below, outbreaks of this pathogen have previously resulted in considerable consumer insecurity, public health challenges, and economic losses for stakeholders, including a multistate listeriosis outbreak associated with cantaloupes grown in Colorado [23] and a listeriosis outbreak of a processed meat product in South Africa [24]. These two outbreaks are considered as one of the largest in the United States, and the largest recorded foodborne outbreak globally, respectively.

From a public health perspective, and from an economic standpoint, *L. monocytogenes* is a very important pathogen. Among the 15 foodborne pathogens that are included in the United States Department of Agriculture Economic Research Service (USDA ERS) report, this pathogen is ranked third, and is estimated to cause an economic burden of $2.8 billion in a typical year in the United States [25]. This includes $600 in annual economic burden associated with prenatal and newborn infections, $510 million of burden for stillbirths and neonatal death, and an additional burden due to disabilities caused by Listeriosis [25]. The disability-adjusted life years (DALYs) for *L. monocytogenes* from domestically acquired foodborne illnesses is 4400 years (90% credible interval; ranging from 1500 to 8400 years) for pregnancy-associated cases, whereas the estimated mean DALYs for non-pregnancy-associated cases is similarly 4400 years (90% credible interval; ranging from 300 to 13,100) [26]. Epidemiological estimates derived from active surveillance data of CDC illustrates that annually, 1455 illness episodes occur in the United States, and the hospitalization and death rates of the pathogen are additionally estimated to be 94.0, and 15.9%, respectively [27]. The pathogen has been involved in at least 81 single and multistate outbreaks from 1998 to 2017—these outbreaks have led to 945, 691, and 140 illness, hospitalization, and death episodes, respectively [6].

In addition to the epidemiological burden of the pathogen, the extreme survival capability of *L. monocytogenes* in environmental samples observed in this study is of great public health significance and is in harmony with previous publications. The pathogen is considered as a ubiquitous microorganism with the ability to survive low pHs, refrigeration, as well as high concentrations of salt [28], and have been previously isolated in high frequencies from surface waters in North America [7]. Although some studies tend to disassociate the risk of listeriosis from surface water [29], the prevalence of this pathogen in surface water in North America, the persistence of the pathogen in surface water illustrated in the current study, and the above-mentioned outbreak of this pathogen associated with a raw agricultural commodity in the United States, highlights the needs for best practices and preventive measures for control of this ubiquitous and opportunistic pathogen in water supplies. 

The results of our trials demonstrate that the persistence of each of the Gram-negative pathogens at this temperature was similar, while differing to a great extent when compared to *L. monocytogenes*. Counts of Shiga toxin-producing *E. coli* O157:H7 (*E. coli* O157:H7) were 4.99 ± 0.3 log CFU/mL and were reduced (*p* < 0.05) to 4.45 ± 0.1 and 4.27 ± 0.0 after one- and two-week aerobic storage at 5 °C (Figure 2A). These respective 0.54 and 0.72 log reductions of *E. coli* O157:H7 were similar to those observed for non-typhoidal *Salmonella* serovars (*Salmonella* serovars). Similar to *E. coli* O157:H7, *Salmonella* serovars log reductions were 0.84 and 1.41 log CFU/mL after one and two weeks of storage, respectively (Figure 2A). Although these two Gram-negative pathogens of the *Enterobacteriaceae* family are not considered to be able to multiply at this low temperature [22], our study indicates that these pathogens were able to survive at this low temperature at a rate that has considerable public health significance. After a 28-day trial, *E. coli* O157:H7 counts were 2.58 ± 0.1 and 1.97 ± 0.1 log CFU/mL (Figure 2A). It is important to note that 2 log CFU/mL of pathogen is equivalent to 100 pathogenic cells per mL, and studies indicate fewer than 1000 cells of Shiga toxin-producing *E. coli* O157:H7, and 10 to 1000 cells of non-typhoidal *Salmonella* serovars, are capable of causing potential human health complications, including Hemolytic Uremic Syndrome and Reactive Arthritis, if ingested [22,30].

Various serovars of *Salmonella* and *E. coli* O157:H7 are also important contributors to the economic burden of foodborne and waterborne diseases. Based on the above-referenced ERS report, non-typhoidal *Salmonella* serovars are ranked first among the dominant foodborne pathogens in the U.S. for causing the highest economic burden. This is estimated to be $3.7 billion on a typical year in the United States with almost 90%, 8%, and 2% of the burden associated with deaths, hospitalizations, and non-hospitalization cases of the pathogen, respectively [25]. Every year, roughly one million Americans experience illness with non-typhoidal *Salmonella* serovars. The Salmonellosis infection has a 27.2% hospitalization rate and around 0.5% death rate (378 annual cases in a typical year) [27]. The estimated mean DALYs for non-typhoidal *Salmonella* serovars is 32,900 (90% credible interval; range from 19,200 to 52,800) [26]. From 1998 to 2017, there had been at least 3467 reported single or multistate outbreaks associated with non-typhoidal *Salmonella* serovars, leading to 83,061, 10,335, and 127 episodes of illness, hospitalization, and death, respectively [6].

The estimated mean DALYs for *E. coli* O157:H7 is 1200 (90% credible interval; range from 540 to 2600), with 68% of the cases from foodborne related illnesses [26]. It is noteworthy that 760 and 460 of the DALYs for this pathogen were associated with Acute Gastroenteritis and Hemolytic Uremic Syndrome, two main sequelae of infections with Shiga toxin-producing *Escherichia coli*. These estimates are currently not available for non-O157 serogroups of the pathogen [26]. From 1998 to 2017, there were at least 717 single and multistate episodes associated with *E. coli* O157:H7, leading to 10,596, 2359, and 3903 episodes of illness, hospitalization, and death, respectively [6]. Active surveillance data of CDC also reveals 63,153 cases of illness with 46.2% and 0.5% hospitalization and death rates, respectively, every year in the United States [27].

In addition to the fate of these pathogens during storage that are articulated in this study, pathogenicity of the pathogens could also be affected by prolonged storage. Assays to determine the motility of these pathogens before and after prolonged storage and reverse transcriptase PCR-based trials to study the expression of virulence genes of these pathogens could be public health significant follow-up experiments for future investigators.

### 3.2. Fate of Planktonic Pathogens at 25 and 37 °C

The Gram-positive pathogen included in the trials, *L. monocytogenes*, belongs to Listeriaceae family that had been taxonomically formed recently [31]. While other two pathogens (*Salmonella* serovars and *E. coli* O157:H7) are Gram-negative and taxonomically different (belonging to Enterobacteriaceae family), all three pathogens exhibited comparable fate and proliferation in surface water at 25 and 37 °C, during the 28-day trials. At 25 °C and on day 0, the counts of *L. monocytogenes*, *E. coli* O157:H7, and *Salmonella* serovars were 4.73 ± 0.1, 5.01 ± 0.1, and 4.76 ± 0.2 log CFU/mL, respectively (Figure 2B). After one-week of static aerobic storage, these counts were similar among the three pathogens (*p* ≥ 0.05) and were 6.12 ± 0.3, 6.06 ± 0.3, and 5.47 ± 0.1, respectively. After four weeks, these counts remained similar, and were 5.39 ± 0.1, 4.94 ± 0.2, 4.94 ± 0.2, respectively (Figure 2B). This indicates the importance of preventive measures for safeguarding the food and water supplies against these pathogens since their fate in surface water at this temperature could exceed several weeks. 

In harmony with the obtained results at ambient temperature, at 37 °C, the pathogens similarly survived for an extended period of time in surface water. The counts on day 0 were 4.68 ± 0.1, 4.90 ± 0.1, and 4.80 ± 0.0 log CFU/mL for *L. monocytogenes*, *E. coli* O157:H7, and *Salmonella* serovars, respectively (Figure 2C). These counts remained similar (*p* ≥ 0.05) after one week of aerobic storage at this temperature and were 5.39 ± 0.1, 5.53 ± 0.1, and 5.42 ± 0.1, respectively. These values remained at their existing level (*p* ≥ 0.05) after the 28-day trial and were 5.09 ± 0.2, 5.18 ± 0.1, and 4.16 ± 0.4, respectively (Figure 2C). 

These results are of great public health significance for assuring the safety of raw agricultural commodities that have no or minimal treatments by stakeholders and consumers before consumption. Our study indicates that the occurrence of contamination with these pathogens could lead to prolonged survival of these microorganisms in low-nutrient environments and highlights the need for preventive measures, such as those articulated under the Produce Safety Rule of the U.S. Food Safety Modernization Act (FSMA).

Additionally, in the landscape of climate change, there is the expectation that there will be considerably more extreme weather events [3]. These extreme events have been associated with the augmentation of the spread of microbial pathogens from animal food production facilities to surface water, that could ultimately pose a public health risk for consumers of raw agricultural commodities. Considering the results of the current study that illustrate the persistence of these pathogens, and considering that climate change will be a “threat multiplier” for the spread of these pathogens, evidence-based mitigation and adaption best practices and legislations, such as FSMA, could assure the safety of food and water supplies in the remainder of the 21st century, under the landscape of the changing climate. 

### 3.3. Biofilm Formation of Non-Typhoidal Salmonella at 25 and 37 °C

Among various thermal and non-thermal procedures common in manufacturing to assure the safety of processed commodities, the application of elevated hydrostatic pressure is gaining increasing momentum in private industry due to advancements in engineering of pressure-based pasteurizers [13,14,15,16]. This technology exposes the final products to elevated hydrostatic pressure to assure safety against pathogenic and spoilage organisms. While this technology is efficacious and validated against an array of pathogenic and spoilage microorganisms, very limited pieces of information are currently available on the persistence and multiplication efficacy of pressure-stressed pathogens, e.g., those that survive a pressure-based pasteurization with sub-lethal injuries. Thus, in addition to wild-type phenotypes, this study further investigated the fate and biofilm formation of pressure-stressed non-typhoidal *Salmonella* serovars. To the best knowledge of the study authors, this is one of the first conducted trials in peer-reviewed literature comparing biofilm formation of wild-type and pressure-stressed *Salmonella* serovars on two abiotic surfaces.

**Figure 3:**
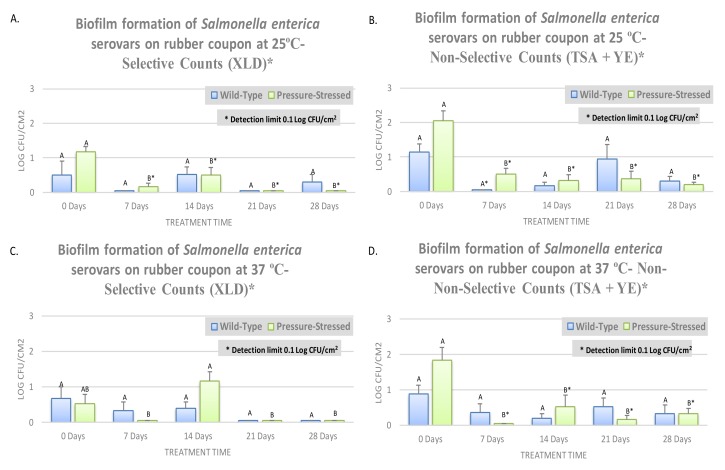
Survival and biofilm formation of a five-strain cocktail of *Salmonella* serovars (ATCC® numbers 13076, 8387, 6962, 9270, 14028), wild-type, and treated by elevated hydrostatic pressure (pressure-stressed) at 15,000 PSI (Barocycler Hub880, Pressure BioScience Inc., South Easton, MA, USA) for 15 min on rubber coupons in autoclaved Cumberland river water for 0, 7, 14, 21, and 28 days at 25 and 37 °C. In each graph, each column of each time interval followed by different uppercase letters are representing log CFU/cm^2^ values that are statistically (*p* < 0.05) different (Tukey-adjusted ANOVA). Uppercase letters followed by * sign are statistically (*p* < 0.05) different than the untreated control (Dunnett’s-adjusted ANOVA). The detection limit on both selective (XLD) and non-selective (TSA + YE) media was 0.1 log CFU/cm^2^. (**A**) Survival of biofilm cells on XLD at 25 °C; (**B**) Survival of biofilm cells on TSA + YE at 25 °C; (**C**) Survival of biofilm cells on XLD at 37 ℃; (**D**) Survival of biofilm cells on TSA + YE at 37 °C.

**Figure 4:**
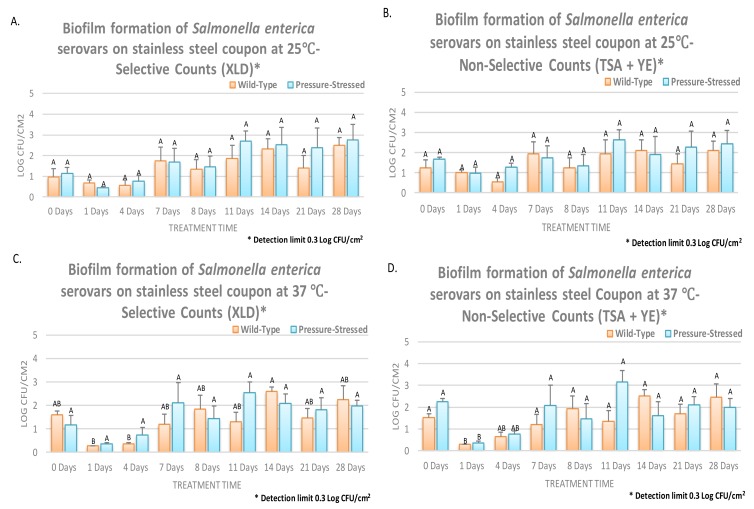
Survival and biofilm formation of five-strain cocktail of *Salmonella* serovars (ATCC® numbers 13076, 8387, 6962, 9270, 14028), wild-type, and treated by elevated hydrostatic pressure (pressure-stressed) at 15,000 PSI (Barocycler Hub880, Pressure BioScience Inc., South Easton, MA, USA) for 15 min on stainless steel coupons in autoclaved Cumberland river water for 0, 1, 4, 7, 8, 11, 14, 21, and 28 days at 25 and 37 °C. In each graph, each column of each time interval is representing log CFU/cm^2^. The detection limit on both selective (XLD) and non-selective (TSA + YE) media was 0.3 log CFU/cm^2^. (**A**) Survival of biofilm mass on XLD at 25 ℃; (**B**) Survival of biofilm mass on TSA + YE at 25 °C; (**C**) Survival of biofilm mass on XLD at 37 °C; (**D**) Survival of biofilm mass on TSA+ YE at 37 ℃.

Similar to trials of planktonic cells, sessile biofilm cells were grown and studied for 28 days on the surfaces of rubber (Figure 3) and stainless steel (Figure 4) coupons. As mentioned above, the trials involved two phenotypes (wild-type and pressure-stressed), were conducted at two temperatures (25 and 37 °C), and enumerations were conducted on surfaces of selective (XLD) and non-selective (TSA + YE) media. 

Overall, consistent in the vast majority of examined phenotypes, enumeration days, and abiotic surfaces, counts of selective medium were lower than those obtained from the non-selective medium (Figure 3 and Figure 4). This is in harmony with previous literature [13,17] and is attributed to the presence of selective and differential agents in the selective medium that inhibits the multiplication of microbial pathogens. At 25 °C and on the surface of rubber, non-selective biofilm counts of wild-type and pressure-stressed *Salmonella* serovars were 1.26 ± 0.4 and 1.66 ± 0.4 log CFU/cm^2^, respectively. The biofilm mass of the *Salmonella* serovars, even in the absence of main micro- and macronutrients, remained constant (*p* ≥ 0.05) and detectable after one, two, three, and four weeks of aerobic storage at 25 °C (Figure 3A,B). These counts were 2.11 ± 0.5 and 2.44 ± 0.7 log CFU/cm^2^ on day 28 of storage for wild-type and pressure-stressed *Salmonella* serovars, respectively (Figure 3A,B). It is noteable that pressure-stressed phenotype at this temperature exhibited comparable biofilm formation and fate relative to the wild-type pathogen, indicating that survivors of exposure to hydrostatic pressure do not pose a risk greater than the wild-type cells, and if the decontamination and sanitation program is validated against the wild-type pathogen, it could almost certainly eliminate the pressure-stressed phenotype as well. At 25 °C and on the surface of stainless steel, both phenotypes also exhibited similar capability for attachment and formation of biofilm. The non-selective biofilm counts of wild-type *Salmonella* serovars on days 0, 7, 14, and 28, on surface of stainless steel coupons, were 1.26, 1.93, 2.12, and 2.11 log CFU/cm^2^, respectively (Figure 3A,B). These counts for the pressure-stressed phenotypes were 1.66, 1.73, 1.92, and 2.44 log CFU/cm^2^, respectively (Figure 4A–D). This further illustrates that both phenotypes have comparable biofilm formation capabilities and that the pressure-stressed phenotype does not pose a risk greater than the wild-type pathogen. In harmony with data obtained from the rubber coupons, selective counts of both phenotypes obtained from stainless steel coupons were lower than those obtained from the yeast extract supplemented non-selective medium (Figure 4A–D).

At 37 °C and on the surface of rubber coupons, selective counts of wild-type *Salmonella* serovars were 1.61 ± 0.2, 1.19 ± 0.4, 2.59 ± 0.2, 2.25 ± 0.6, and 1.46 ± 0.4 log CFU/cm^2^ on days 0, 7, 14, 21, and 28, respectively (Figure 3D). These counts were similarly 1.16 ± 0.4, 2.12 ± 0.9, 2.09 ± 0.4, 1.82 ± 0.5, and 1.97 ± 0.3 log CFU/cm^2^, respectively, for the pressure-stressed phenotype of *Salmonella* serovars (Figure 3C). Non-selective counts exhibited similar trends with slightly higher values, as delineated earlier in this section. As an example, non-selective biofilm counts of wild-type and pressure-stressed pathogens were 2.45 ± 0.6 and 1.98 ± 0.4 log CFU/cm^2^ on day 28 on rubber coupons (Figure 3D). These non-selective biofilm counts exhibited similar trends on the surface of stainless steel where 1.51, 1.20, 2.52, 1.71, and 2.45 log CFU/cm^2^ of wild-type *Salmonella* serovars and 2.25, 2.07, 1.60, 2.12, and 1.98 log CFU/cm^2^ of pressure-stressed *Salmonella* serovars were observed on days 0, 7, 14, 21, and 28, respectively (Figure 4B,D).

These findings are particularly important since previous literature indicates that contamination of surface water could transfer a pathogen to raw agricultural commodities [32]. While some studies only discuss the transfer of pathogens to the surface of a crop, others indicate enteric and pathogenic bacteria could become internalized in edible and non-edible tissues of crops after exposure to contaminated water before harvest and during primary and further processing [33,34,35]. Formation of bacterial biofilm had also been associated with the accelerated persistence and transfer of pathogens to a crop [36].

Results of these biofilm assays indicate that not only are pathogenic cells in the planktonic stage capable of prolonged survival in surface water, but that they have the capability to form and maintain biofilm masses on surfaces of both rubber and stainless steel. These findings emphasize the need for the incorporation of preventive measures for assuring the safety of surface water, raw agricultural commodities, and those that are considered ready-to-eat, since the occurance of contamination in growing, harvesting, and packaging areas could lead to prolong the persistence of the pathogens in the environment in both planktonic and biolfim forms.

## 4. Conclusions

Under the conditions of our experiments, we observed a prolonged persistence of *L. monocytogenes*, Shiga toxin-producing *E. coli* O157:H7, and non-typhoidal *Salmonella* serovars in surface water during the 28-day trials. While *L. monocytogenes* exhibited a lower survival rate at 5 °C relative to the Gram-negative pathogens, at higher temperatures of 25 and 37 °C, all three pathogens exhibited similar trends for survival in surface water. This prolonged survival rate is of particular importance from a public health perspective for assuring the safety of surface waters and raw agricultural commodities and those that are considered ready-to-eat. The survival of the pathogens for four weeks in the tested low-nutrient environment emphasizes the need for preventive measures, such as those articulated in Produce Safety Rule of Food Safety Modernization Act, to minimize the risk of contamination in production and processing environments. Persistence of these bacterial pathogens in low-nutrient environments is of great public health significance for the purification of municipal waters, for maintaining microbiological safety of recreational waters, and for the safety of raw agricultural commodities that are in direct contact with surface and sub-surface waters before and after harvest. These findings have even greater implications for regions of the world with sub-optimal processes for treatment of municipal and agricultural wastewaters and for sanitization of surface and sub-surface waters for drinking and industrial applications. 

Non-typhoidal *Salmonella* serovars additionally exhibited the capability to form and maintain biofilm masses on the surface of rubber and stainless steel. Pressure-stressed phenotype of the pathogen was also efficacious in the formation and maintenance of sessile cell masses on the above-mentioned abiotic surfaces at a rate that was comparable to wild-type pathogen. This indicates that pressure-stressed phenotype of the pathogen could be controled with an intervention validated against wild-type *Salmonella* serovars.

Climate change, as a “threat multiplier” for the spread of infectious diseases, will undoubtably lead to increased extreme weather events, such as more frequent flooding, and in part, could lead to contamination of drinking and agricultural waters by enteric and pathogenic bacteria originating from animal food production. Considering the results of our study, delineating the capability of the pathogens for prolonged survival and proliferation in surface water, the development of evidence-based mitigation and adaption programs are unequivocally needed for further assuring the safety of our food and water supplies in the landscape of changing climate.
